# Sociodemographic disparities in influenza vaccination among older adults in United States

**DOI:** 10.3389/fpubh.2025.1474677

**Published:** 2025-02-07

**Authors:** Huan Tao, Jin Chen, Xue Zhang, Tao Wang, Nenggang Jiang, Yongqian Jia

**Affiliations:** ^1^Department of Hematology, West China Hospital of Sichuan University, Chengdu, China; ^2^Department of Evidence-based Medicine and Clinical Epidemiology, West China Hospital of Sichuan University, Chengdu, China; ^3^State Key Laboratory of Experimental Hematology, National Clinical Research Center for Blood Diseases, Haihe Laboratory of Cell Ecosystem, Institute of Hematology and Blood Diseases Hospital, Chinese Academy of Medical Sciences and Peking Union Medical College, Tianjin, China; ^4^Tianjin Institutes of Health Science, Tianjin, China; ^5^Department of Laboratory Medicine, West China Hospital of Sichuan University, Chengdu, China

**Keywords:** influenza vaccine, racial groups, socioeconomic disparities, older adults, public health

## Abstract

**Background:**

Influenza vaccination uptake among United States adults aged 65 years or older remains suboptimal and stagnant. This study aims to evaluate the prevalence of influenza vaccination and examine sociodemographic disparities within a nationally representative sample.

**Methods:**

This study is a cross-sectional study. We used the data from the Behavioral Risk Factor Surveillance System spanning the years 2011 to 2022. Logistic regression models were used to assess potential associations between influenza vaccination uptake and sociodemographic characteristics. Concentration indexes were also calculated to measure the socioeconomic inequalities on influenza vaccination uptake.

**Results:**

The study included 1,391,440 adults aged 65 years and older, with 62.87% reporting having received an influenza vaccination. The weighted prevalence of influenza vaccination uptake showed a slight increase, ranging from 59.05% in 2011–2013 to 67.49% in 2020–2022. Higher vaccination rates were observed among non-Hispanic Whites [63.16%; odds ratio (OR) 1.38, (95% CI 1.33–1.42)], individuals with education above high school [63.89%; OR 1.16, (95% CI 1.12–1.19)], and those with an income above $50,000 [65.86%; OR 1.47, (95% CI 1.43–1.50)]. Compared to non-Hispanic Black people with an income below $25,000 and education less than high school, the ORs were significantly higher among non-Hispanic whites [2.12, (95% CI 1.97–2.28)], non-Hispanic Black people [1.30, (95% CI 1.18–1.44)], and Hispanics [1.40, (95% CI 1.24–1.59)] earning above $50,000 and education above high school. Those who received an influenza vaccination tended to be concentrated in the high-income group and high-education group.

**Conclusion:**

There are substantial racial and socioeconomic disparities in influenza vaccination uptake among individuals aged 65 years or older. Health policy maybe urgently needed to reduce these avoidable inequalities.

## 1 Introduction

Influenza viruses circulate annually within the United States, and annual administration of the influenza vaccination is recommended for adults aged 65 years or older, who are at high risk of serious illness, hospitalization, and death due to influenza. This age group accounts for most influenza-related deaths and hospital admissions (1, 2). Moreover, data from the Hospitalized Adult Influenza Vaccine Effectiveness Network indicates a decline in vaccine effectiveness of approximately 10–11% per month specifically in individuals aged 65 years or older (3). Understanding influenza vaccination uptake in these vulnerable populations may inform future public health strategies.

Previous studies suggested that influenza vaccination uptake and influenza hospitalization rates differed by race/ethnicity (4). And individuals with higher socioeconomic status were more likely to receive an influenza vaccination (5). However, the evidence regarding sociodemographic disparities in influenza vaccination uptake, and the potential joint associations between socioeconomic inequalities and race/ethnicity in influencing these rates across the United States, is still not sufficiently comprehensive. The insights gained from these findings could help identify influenza vaccination disparities throughout the United States and offer guidance for policymakers in striving for a more inclusive and equitable allocation of resources.

Thus, this study aimed to compare the weighted and age-standardized prevalence of influenza vaccination uptake between United States adults aged 65 years or older from 2011 to 2022, and to assess the socioeconomic disparities and their potential joint association on overall influenza vaccination uptake using a nationally representative sample.

## 2 Methods

### 2.1 Data

We included individuals aged 65 years or older, utilizing data from the Behavioral Risk Factor Surveillance System (BRFSS) spanning the years 2011 to 2022 (6). The BRFSS, funded by the Centers for Disease Control and Prevention (CDC), is an annual survey designed to gather comprehensive information on sociodemographic factors, health behaviors, and comorbid conditions among non-institutionalized adults residing in the United States, Guam, and Puerto Rico. As the largest annual health survey across the 50 states, BRFSS enables estimates of Medicare’s effects at age 65 years at national and state levels. Given that BRFSS data are publicly accessible, this study was exempt from review by the institutional review board committee.

### 2.2 Variables

Influenza vaccination uptake was self-reported, and individuals deemed to have received the influenza vaccination if they responded affirmatively to having been vaccinated within the 12 months preceding the survey completion. Age and sex were assessed via survey questions. Race/ethnicity was self-reported, with race categorized as non-Hispanic White, non-Hispanic Black, Hispanic, and other races. Education level was assessed by asking participants, “What is the highest grade or year of school you have completed?” and categorized as less than high school, high school, and above high school. Income was assessed via the question, “Is your annual income from all sources: less than $10, 000, or $10,000 to less than $15,000, or $15,000 to less than $20,000, or $20,000 to less than $25,000, or $25,000 to less than $35,000, or $35,000 to less than $50,000, or $50,000 to less than $75,000, or $75,000 or more.” Annual family income was then categorized as less than $25,000, $25,000 to $50,000, and above $50,000.

### 2.3 Statistical analysis

The weighted prevalence of influenza vaccination uptake among individuals in the United States aged 65 years or older from 2011 to 2022 was calculated based on the survey weights, as the BRFSS utilized design weighting (7). We used 2010 US Census population proportions to calculate the age-standardized prevalence of influenza vaccination uptake (8). Trends in prevalence over time were tested using a weighted logistic regression model.

For descriptive purposes, continuous data was grouped into categorical data, and categorical data are presented as numbers and percentages. Since the influenza vaccination uptake was a binary variable, thus adjusted odds ratios (ORs) with 95% confidence interval (CI) were calculated using binary logistic regression models to assess potential associations between influenza vaccination uptake and sociodemographic characteristics. Interactions between influenza vaccination uptake and socioeconomic factors were tested for predicting receipt of each vaccine and reported if statistically significant. Concentration indexes were calculated to measure socioeconomic inequality, with values ranging from −1 to +1. Positive (negative) values indicate that education or income is concentrated among rich (poor) individuals. A concentration index of zero indicates no inequality. All *p*-values were two-sided, with a significance level of 0.05. Analysis was performed using Stata version 15.0 (Stata Corp), and weighting procedures accounted for the complex survey design of the BRFSS.

## 3 Results

### 3.1 Crude prevalence of influenza vaccination uptake

The study included 1,391,440 older adults individuals aged 65 years and above, with a gender distribution of 593,748 males (42.67%) and 797,692 females (57.33%). Among these, 878,842 individuals (63.16%) had an education level above high school, and 528,726 individuals (38.00%) earned above $50,000 annually. Ethnically, the sample consisted of 1,189,024 White individuals (85.45%), 86,600 Black people (6.22%), 54,890 Hispanics (3.94%), and 60,926 individuals of other races (4.38%). Nearly two-thirds (874,863, 62.87%) reported receiving an influenza vaccination (detailed in Table 1).

**Table 1:** Prevalence and distribution of influenza vaccination by patient characteristics among individuals aged 65 years or more based on BRFSS database from 2011 to 2022.

Characteristics	Subgroups	No. vaccinated/total ^a^	% Vaccinated (95% CI)^b^	OR (95%CI) ^c^	Std. error	*t* value	*P* value
Overall	Crude	874,863 (1,391,440)	61.24 (61.05–61.42)				
	Age-standardization		61.65 (61.47–61.84)				
Age, y							
	65–69	265,076 (456,657)	56.33 (56.00–56.65)	1 [Reference]			
	70–74	233,655 (371,578)	61.71 (61.35–62.06)	1.27 (1.24–1.29)	0.013	23.07	<0.001
	75–79	171,246 (261,061)	64.32 (63.89–64.74)	1.47 (1.43–1.50)	0.017	32.47	<0.001
	≥80	204,886 (302,144)	65.77 (65.36–66.18)	1.62 (1.59–1.66)	0.019	41.19	<0.001
Gender							
	Male	373,971 (593,748)	61.10 (60.82–61.38)	1 [Reference]			
	Female	500,892 (797,692)	61.36 (61.11–61.61)	1.05 (1.03–1.07)	0.009	5.82	<0.001
Education							
	<High School	61,199 (109,281)	55.38 (54.74–56.02)	1 [Reference]			
	High School	240,905 (403,317)	58.94 (58.61–59.27)	1.00 (0.98–1.04)	0.015	0.32	0.75
	>High School	572,759 (878,842)	63.89 (63.66–64.12)	1.16 (1.12–1.19)	0.018	9.50	<0.001
Income							
	<$25,000	241,070 (423,012)	55.60 (55.25–55.96)	1 [Reference]			
	≥$25,000 and<$50,000	275,271 (439,702)	61.05 (60.73–61.38)	1.18 (1.16–1.21)	0.012	15.51	<0.001
	≥$50,000	358,522 (528,726)	65.86 (65.57–66.15)	1.47 (1.43–1.50)	0.017	33.81	<0.001
Race							
	Non-Hispanic White	763,638 (1,189,024)	63.16 (62.98–63.34)	1.38 (1.33–1.42)	0.022	19.75	<0.001
	Non-Hispanic Black	46,665 (86,600)	52.86 (52.12–53.60)	1 [Reference]			
	Hispanic	28,777 (54,890)	53.35 (52.40–54.29)	1.14 (1.08–1.19)	0.029	5.11	<0.001
	Other	35,783 (60,926)	59.22 (57.92–60.52)	1.26 (1.18–1.34)	0.040	7.16	<0.001

a. Unweighted.

b. Weighted percentage and 95%CI.

c. Model adjusted for age, sex, income, education and race/ethnicity.

The weighted prevalence of influenza vaccination exhibited a slight increase over the study period, ranging from 59.05% in 2011–2013 to 67.49% in 2020–2022. This increase was consistent across age, sex, education, income and race subgroups (detailed in Supplementary Table 1). Individuals aged 80 years or older [OR 1.63, (95%CI, 1.59–1.66)] were more likely to receive an influenza vaccination, followed by those aged 75 to 79 years [OR 1.47, (95%CI, 1.43–1.50)] and those aged 70 to 74 years [OR 1.27, (95%CI, 1.24–1.29)] compared to those aged 65 to 69 years. Respondents with education above high school [63.89%; OR 1.16, (95% CI, 1.12–1.19)] were more likely to receive an influenza vaccination than those with less education (55.38%). Individuals earning above $50,000 [65.86%; OR 1.47, (95% CI, 1.43–1.50)] and those earning between $25,000 and $50,000 [61.05%; OR 1.18, (95% CI, 1.16–1.21)] had higher rates of influenza vaccination uptake than those earning less than $25,000 (55.60%). Non-Hispanic White individuals [63.16%; OR 1.38, (95% CI, 1.33–1.42)] and Hispanic individuals [53.35%; OR 1.14, (95% CI, 1.08–1.19)] were more likely to have been vaccinated than Black individuals (52.86%).

### 3.2 Age-standardized rates of influenza vaccination uptake varied in socioeconomic factors by races

Using the 2010 U.S. Census as the reference population, we calculated age-standardized rates of influenza vaccination uptake (Table 2). There were noticeable increases in age-standardized rates of influenza vaccination uptake for non-Hispanic White and Black individuals with income above $50,000 than those with income less than $25,000, varied in education levels non-Hispanic White (increased by 1.27% for less than a high school, 5.37% for a high school, and 12.38% for above high school); non-Hispanic Black (increased by −7.42% for less than a high school, 3.90% for a high school, and 8.34% for above high school). The increase was more pronounced from 2020 to 2022 (detailed in Supplementary Table 2).

**Table 2:** Age-standardized rates of influenza vaccination uptake varied in socioeconomic factors by races.

Survey years	Races subgroups			Income level	
			Less than $25,000	$25,000 to less than $50,000	$50,000 or more
		Education level	Proportion (%) 95%CI	Proportion (%) 95%CI	Proportion (%) 95%CI
Overall	**Non-Hispanic White**				
		<High School	56.15 (55.24–57.05)	57.73 (56.33–59.11)	57.42 (54.98–59.82)
		High School	57.30 (56.77–57.84)	61.52 (61.00–62.04)	62.67 (61.89–63.45)
		>High School	57.39 (56.81–57.96)	63.82 (63.41–64.22)	69.77 (69.45–70.07)
	**Non-Hispanic Black**				
		<High School	52.36 (50.50–54.22)	54.22 (50.15–58.25)	44.94 (37.31–52.82)
		High School	50.89 (49.23–52.54)	53.52 (51.14–55.88)	54.79 (49.81–59.67)
		>High School	50.12 (48.07–52.16)	54.04 (52.11–55.96)	58.46 (56.02–60,85)
	**Hispanic**				
		<High School	52.87 (51.17–54.57)	53.21 (48.15–58.20)	58.30 (47.72–68.16)
		High School	51.84 (49.50–5.17)	55.11 (51.40–58.75)	58.05 (52.37–63.52)
		>High School	50.29 (47.70–52.87)	56.63 (53.59–59.63)	60.46 (57.77–63.08)
	**Other**				
		<High School	52.89 (49.05–56.69)	54.79 (48.45–60.97)	54.64 (42.37–66.27)
		High School	55.78 (52.13–59.38)	59.55 (54.42–64.48)	63.22 (56.26–69.67)
		>High School	55.96 (52.80–59.07)	59.54 (56.11–62.89)	64.76 (62.17–67.26)

### 3.3 Joint associations of socioeconomic factors and races with influenza vaccination uptake

After adjusting for age and sex, the adjusted ORs were calculated to compare the likelihood of influenza vaccination uptake among racial and socioeconomic groups (detailed in Table 3). Compared to non-Hispanic Black individuals with an income less than $25,000 and education less than high school, the ORs were significantly higher among all non-Hispanic White groups (ORs ranging from 1.15 to 2.12), non-Hispanic Black individuals with income above $50,000 and education above high school (1.30, 95% CI, 1.18–1.44), and some Hispanic groups with higher income and higher education (Statistical test parameters were detailed in Supplementary Table 3).

**Table 3:** Joint associations between socioeconomic factors and races in predicting the influenza vaccination uptake.

Income level							
Races	Education	Less than $25,000		$25,000 to less than $50,000		$50,000 or more	
		OR (95%CI)	*p* value	OR (95%CI)	*p* value	OR (95%CI)	*p* value
Non-Hispanic White							
	<High school	1.15 (1.06–1.24)	0.001	1.25 (1.14–1.37)	<0.001	1.24 (1.09–1.40)	0.001
	High school	1.20 (1.12–1.30)	<0.001	1.44 (1.33–1.55)	<0.001	1.51 (1.40–1.64)	<0.001
	>High school	1.22 (1.13–1.31)	<0.001	1.60 (1.48–1.72)	<0.001	2.12 (1.97–2.28)	<0.001
Non-Hispanic Black							
	<High school	Refer.		1.06 (0.88–1.28)	0.527	0.77 (0.55–1.07)	0.118
	High school	0.94 (0.85–1.03)	0.183	1.05 (0.94–1.18)	0.378	1.07 (0.88–1.29)	0.49
	>High school	0.92 (0.83–1.03)	0.131	1.09 (0.98–1.20)	0.11	1.30 (1.18–1.44)	<0.001
Hispanic							
	<High school	1.06 (0.96–1.17)	0.214	1.15 (0.94–1.40)	0.167	1.58 (1.10–2.27)	0.014
	High school	1.01 (0.90–1.14)	0.832	1.17 (1.00–1.37)	0.057	1.26 (0.99–1.60)	0.058
	>High school	0.94 (0.83–1.06)	0.316	1.22 (1.05–1.40)	0.007	1.40 (1.24–1.59)	<0.001
Other							
	<High school	1.05 (0.88–1.25)	0.579	1.12 (0.83–1.51)	0.453	1.29 (0.66–2.51)	0.452
	High school	1.25 (1.06–1.46)	0.006	1.35(1.09–1.67)	0.007	1.57 (1.13–2.17)	0.006
	>High school	1.21 (1.05–1.39)	0.010	1.46 (1.25–1.70)	<0.001	1.78 (1.57–2.01)	<0.001

### 3.4 The concentration indexes

Overall, those who received an influenza vaccination tended to be concentrated in the high-income group (index value 0.0981 for earning above $50,000, *p* value <0.001) and high-education group (index value 0.0812 for above high school, *p* value <0.001). This trend was consistent among non-Hispanic White, non-Hispanic Black, and Hispanic individuals (detailed in Table 4). The concentration index for income and education showed a notable increase from 2011 to 2022 (income coefficient 0.0077; 95% CI, 0.0047 to 0.0107, and education coefficient 0.0048; 95% CI, 0.0028 to 0.0068) (detailed in Table 5).

**Table 4:** Concentration index among different income and education levels by races.

Characteristics		Subgroups	No.	Index value	Std. error	*P* value
Overall			1,391,440	0.0650	0.0010	<0.001
	Income	<$25,000	423,012	0.0037	0.0018	0.04
		≥$25,000 and<$50,000	439,702	0.0388	0.0018	<0.001
		≥$50,000	528,726	0.0981	0.0017	<0.001
	Education	<High school	109,281	0.0067	0.0035	0.06
		High school	403,317	0.0225	0.0018	<0.001
		>High school	878,842	0.0812	0.0013	<0.001
Non-Hispanic White						
	Income	<$25,000	324,607	−0.0038	0.0020	0.07
		≥$25,000 and<$50,000	384,506	0.0399	0.0019	<0.001
		≥$50,000	479,911	0.1002	0.0018	<0.001
	Education	<High school	70,130	−0.0125	0.0044	0.0045
		High school	345,615	0.0219	0.0020	<0.001
		>High school	773,279	0.0830	0.0014	<0.001
Non-Hispanic Black						
	Income	<$25,000	41,813	0.0491	0.0056	<0.001
		≥$25,000 and<$50,000	24,849	0.0746	0.0073	<0.001
		≥$50,000	19,938	0.1218	0.0082	<0.001
	Education	<High school	15,170	0.0432	0.0093	<0.001
		High school	27,264	0.0586	0.0070	<0.001
		>High school	44,166	0.0996	0.0055	<0.001
Hispanic						
	Income	<$25,000	33,524	0.0642	0.0063	<0.001
		≥$25,000 and<$50,000	12,221	0.0536	0.0104	<0.001
		≥$50,000	9,145	0.0936	0.0122	<0.001
	Education	<High school	17,643	0.0834	0.0086	<0.001
		High school	14,540	0.0394	0.0095	<0.001
		>High school	22,707	0.0949	0.0076	<0.001
Other						
	Income	<$25,000	23,068	−0.0273	0.0076	0.0003
		≥$25,000 and<$50,000	18,126	0.0034	0.0087	0.69
		≥$50,000	19,732	0.0672	0.0085	<0.001
	Education	<High school	6,338	−0.0477	0.0145	0.001
		High school	15,898	−0.0135	0.0092	0.14
		>High school	38,690	0.0441	0.0060	<0.001

**Table 5:** Trends of concentration indexes over time were tested using logistic regression model.

Characteristics		Subgroups	Coefficient	95%CI		*P* value	*R* ^2^	*F* statistic
				Lower	Upper			
Income	Overall		0.0077	0.0047	0.0107	<0.001	0.7678	33.07
	Age	65–69 years	0.0050	0.0025	0.0075	0.001	0.6675	20.07
		70–74 years	0.0081	0.0050	0.0112	<0.001	0.7716	33.79
		75–79 years	0.0085	0.0045	0.0124	0.001	0.6939	22.66
		80 years or more	0.0063	0.0030	0.0095	0.001	0.6540	18.9
	Sex	Male	0.0084	0.0051	0.0117	<0.001	0.7651	32.57
		Female	0.0073	0.0041	0.0105	<0.001	0.7189	25.57
	Races	Non-Hispanic White	0.0082	0.0058	0.0107	<0.001	0.8467	55.24
		Non-Hispanic Black	0.0055	0.0006	0.0105	0.031	0.3854	6.27
		Hispanic	0.0035	−0.0026	0.0096	0.227	0.1419	1.65
		Other	0.0083	0.0043	0.0122	0.001	0.6845	21.69
Education	Overall		0.0048	0.0028	0.0068	<0.001	0.7387	28.28
	Age	65–69 years	0.0051	0.0033	0.0070	<0.001	0.7916	37.98
		70–74 years	0.0035	0.0019	0.0051	0.001	0.6922	22.49
		75–79 years	0.0038	0.0015	0.0061	0.004	0.5816	13.9
		80 years or more	0.0039	0.0010	0.0069	0.014	0.4713	8.91
	Sex	Male	0.0057	0.0033	0.0081	<0.001	0.7379	28.15
		Female	0.0038	0.0019	0.0057	0.001	0.6689	20.2
	Races	Non-Hispanic White	0.0054	0.0039	0.0068	<0.001	0.8721	68.16
		Non-Hispanic Black	0.0049	0.0009	0.0088	0.021	0.4269	7.45
		Hispanic	0.0021	−0.0036	0.0077	0.437	0.0616	0.66
		Other	0.0063	0.0025	0.0101	0.004	0.5819	13.91

## 4 Discussion

In this nationally representative sample, influenza vaccination uptake rates among individuals aged 65 years or older showed a clear increasing trend from 2011 to 2022. Lower education levels, lower income levels, and non-Hispanic Black individuals were associated with lower influenza vaccination uptake, indicating evident racial and socioeconomic disparities. Non-Hispanic White individuals with any level of socioeconomic status were more likely to receive an influenza vaccination compared to non-Hispanic Black individuals with lower socioeconomic status. Overall, individuals with higher socioeconomic status have greater advantages in influenza vaccination uptake.

Although annual influenza vaccination is recommended for adults aged 65 years or older, the influenza vaccination uptake remains low in previous studies (9, 10). In our study, similarly, only 62.60% individuals reported receiving an influenza vaccination using BRFSS data from 2011 to 2022. Following the release of the first COVID-19 vaccines, individuals who received an influenza vaccination were more likely to report getting a COVID-19 vaccine (11). In our study, the weighted influenza vaccination rate in 2020–2022 was the highest at 67.48%, possibly due to the increasing trend of COVID-19 vaccination.

Low influenza vaccination coverage in U.S. adults suggests that a multitude of factors may be responsible for under-vaccination or non-vaccination including vaccine hesitancy (12). Social media as a source of health information, those users of Twitter and Facebook as sources of health information were more likely to be vaccinated in comparison to users who do not use Twitter or Facebook as a source of health information (13). More than a third of adults were hesitant to receive an influenza vaccination for concerns about vaccination side effects, serious side effects or healthcare provider was not the most trusted source of information about influenza vaccinations (12). Thus, the government departments should strengthen promotional efforts for influenza vaccination on social media platforms. The government departments should intensify their promotional efforts actively on social media platforms to disseminate knowledge related to influenza vaccination.

Racial and ethnic inequities in access and use of the influenza vaccine are pervasive and persistent (14, 15). Previous study indicated that white adults 65 years and older were significantly more likely to receive influenza vaccine than those blacks (16), since that those whites persons were more likely to believe the vaccine is very effective than blacks (66% vs. 50%). Our findings show a 38% higher rate of influenza vaccination among non-Hispanic White individuals and a 14% higher rate among Hispanic individuals compared to Black individuals. Further analysis of joint associations revealed that non-Hispanic White individuals earning above $50,000 with education above high school had a 112% higher rate of receiving an influenza vaccination relative to Black individuals earning less than $25,000 with less than a high school education, a finding rarely described before. Besides, socioeconomic inequalities in access to influenza vaccination uptake are evident (17, 18). Our study indicates that adults who received an influenza vaccination tend to be concentrated in high-income and high-education groups, who have higher health awareness and health literacy, another finding rarely described before.

The principal strength of this study is the use of the BRFSS database, which included 1,391,440 older adults individuals aged 65 years and older spanning the years 2011 to 2022, compared to smaller samples in previous studies based on the BRFSS database (10, 17). Limitations include the reliance on self-reported vaccination history, which may introduce recall bias. This was especially relevant to the pneumococcal vaccine where the respondent was asked to recall whether they ever had received the immunization. While the BRFSS weighting procedures account for the undersampling of populations with reduced telephone coverage and racial minorities, these do not correct for the underrepresentation of people with severe mental, cognitive, and communication limitations. Moreover, BRFSS data do not ask questions surrounding attitudes and beliefs toward vaccines, we were not able to evaluate reasons for opting in or opting out of influenza vaccination. This is a cross-sectional study, and causal relationship cannot be determined even when relevant confounders are adequately controlled. Finally, our results might be subject to cohort and period effects, and further studies are needed to examine the role of vaccine hesitancy or other additional obstacles to vaccination among old adults. Strategies to improve influenza vaccination uptake and completion include disseminating health information through social media, leveraging university or community vaccination campaigns, identifying the need for vaccination, and eliminating barriers.

In conclusion, using the BRFSS 2011–2022 database, older adults with higher socioeconomic status have greater advantages in influenza vaccination uptake. Strategies to increase influenza vaccination uptake in the United States should address barriers faced by people with racial and socioeconomic inequalities. Addressing existing disparities requires attention to the role of social determinants of health in determining access to vaccination, particularly among older people, racial and ethnic minority populations.

## Data Availability

The original contributions presented in the study are included in the article/Supplementary material, further inquiries can be directed to the corresponding author.

## References

[1] Okoli GN Abou-Setta AM Neilson CJ Chit A Thommes E Mahmud SM. Determinants of seasonal influenza vaccine uptake among the elderly in the United States: a systematic review and Meta-analysis. Gerontol Geriatr Med. (2019) 5:2333721419870345. doi: 10.1177/2333721419870345, PMID: 31453267 PMC6698992

[2] Grohskopf LA Blanton LH Ferdinands JM Chung JR Broder KR Talbot HK. Prevention and control of seasonal influenza with vaccines: recommendations of the advisory committee on immunization practices - United States, 2022-23 influenza season. MMWR Recomm Rep. (2022) 71:1–28. doi: 10.15585/mmwr.rr7101a1, PMID: 36006864 PMC9429824

[3] Ferdinands JM Gaglani M Martin ET Monto AS Middleton D Silveira F. Waning vaccine effectiveness against influenza-associated hospitalizations among adults, 2015-2016 to 2018-2019, United States hospitalized adult influenza vaccine effectiveness network. Clin Infect Dis. (2021) 73:726–9. doi: 10.1093/cid/ciab045, PMID: 33462610 PMC8499703

[4] Kawai K Kawai AT. Racial/ethnic and socioeconomic disparities in adult vaccination coverage. Am J Prev Med. (2021) 61:465–73. doi: 10.1016/j.amepre.2021.03.023, PMID: 34334289

[5] Al Rifai M Khalid U Misra A Liu J Nasir K Cainzos-Achirica M. Racial and geographic disparities in influenza vaccination in the U.S. among individuals with atherosclerotic cardiovascular disease: renewed importance in the setting of COVID-19. American journal of. Prev Cardiol. (2021) 5:100150. doi: 10.1016/j.ajpc.2021.100150, PMID: 33521756 PMC7826080

[6] Song S White A Kucik JE. Use of selected recommended clinical preventive services - behavioral risk factor surveillance system, United States, 2018. MMWR Morb Mortal Wkly Rep. (2021) 70:461–6. doi: 10.15585/mmwr.mm7013a1, PMID: 33793461 PMC8022875

[7] CDC, Weighting the BRFSS data 2017, pp. https://www.cdc.gov/brfss/annual_data/2017/ pdf/weighting-2017-508.pdf.

[8] Li C Ford ES Zhao G Wen XJ Gotway CA. Age adjustment of diabetes prevalence: use of 2010 US census data. J Diabetes. (2014) 6:451–61. 24393518 10.1111/1753-0407.12122 PMC11287713

[9] Khan SR Hall AG Tanner RJ Marlow NM. Association between race/ethnicity and disability status and receipt of vaccines among older adults in Florida. Disabil Health J. (2018) 11:339–44. doi: 10.1016/j.dhjo.2017.11.004, PMID: 29198816

[10] Greiner B Hartwell M. Influenza vaccination uptake trends by age, race, and ethnicity in the United States between 2017 and 2020. J Prim Care Community Health. (2022) 13:21501319221104917. doi: 10.1177/21501319221104917, PMID: 35678259 PMC9189513

[11] Davis TC Vanchiere JA Sewell MR Davis AB Wolf MS Arnold CL. Influenza and COVID-19 vaccine concerns and uptake among patients cared for in a safety-net health system. J Prim Care Community Health. (2022) 13:36361. doi: 10.1177/21501319221136361 PMC9716187 36448443

[12] Srivastav A Lu PJ Amaya A Dever JA Stanley M Franks JL. Prevalence of influenza-specific vaccination hesitancy among adults in the United States, 2018. Vaccine. (2023) 41:2572–81. doi: 10.1016/j.vaccine.2023.03.008, PMID: 36907734 PMC10941755

[13] Ahmed N Quinn SC Hancock GR Freimuth VS Jamison A. Social media use and influenza vaccine uptake among White and African American adults. Vaccine. (2018) 36:7556–61. doi: 10.1016/j.vaccine.2018.10.049, PMID: 30389192 PMC10655625

[14] Mahmud SM Xu L Hall LL Puckrein G Thommes E Loiacono MM. Effect of race and ethnicity on influenza vaccine uptake among older US Medicare beneficiaries: a record-linkage cohort study. Lancet Healthy Longevity. (2021) 2:e143–53. doi: 10.1016/S2666-7568(20)30074-X, PMID: 36098112

[15] Grandhi GR Mszar R Vahidy F Valero-Elizondo J Blankstein R Blaha MJ. Sociodemographic disparities in influenza vaccination among adults with atherosclerotic cardiovascular disease in the United States. JAMA. Cardiology. (2020). doi: 10.1001/jamacardio.2020.3978 PMC7489417 32902562

[16] Groom HC Zhang F Fisher AK Wortley PM. Differences in adult influenza vaccine-seeking behavior: the roles of race and attitudes. J Public Health Manag Pract. (2014) 20:246–50. doi: 10.1097/PHH.0b013e318298bd88, PMID: 23715220

[17] Brewer LI Ommerborn MJ Nguyen AL Clark CR. Structural inequities in seasonal influenza vaccination rates. BMC Public Health. (2021) 21:1166. doi: 10.1186/s12889-021-11179-9, PMID: 34140009 PMC8210739

[18] Wallace J Jiang K Goldsmith-Pinkham P Song Z. Changes in racial and ethnic disparities in access to care and health among US adults at age 65 years. JAMA Intern Med. (2021) 181:1207–15. doi: 10.1001/jamainternmed.2021.3922, PMID: 34309621 PMC8314180

