# Supplementary material for: Sociodemographic disparities in influenza vaccination among older adults in United States
Source: Front Public Health. 2025 Feb 7;13:1474677. doi: 10.3389/fpubh.2025.1474677 (PMC11843045; doi:10.3389/fpubh.2025.1474677)
Supplement: Supplementary file 1 [file Table_1.docx]

**Supplement table1 Characteristics of Influenza Vaccination Among Individuals aged 65 years or more among BRFSS database by 2011-2013,2014-2016,2017-2019 and 2020-2022.**

| Characteristics | Subgroups | 2011-2013 | | | 2014-2016 | | 2017-2019 | | 2020-2022 | |
| --- | --- | --- | --- | --- | --- | --- | --- | --- | --- | --- |
|  |  | Vaccinated (%) | Weight%（95%CI） | | Vaccinated (%) | weight%（95%CI） | Vaccinated (%) | weight%（95%CI） | Vaccinated (%) | weight%（95%CI） |
| Overall | Crude | 220688 (60.68) | 59.05 (58.72-59.38) | | 220297 (60.87) | 58.78 (58.44-59.12) | 207456 (60.10) | 59.05 (58.67-59.43) | 226422 (69.27) | 67.49 (67.08-67.89) |
|  | Age-standardization | | 59.37 (59.04-59.70) | |  | 59.28 (58.94-59.63) |  | 59.52 (59.13-59.91) |  | 67.86 (67.45-68.28) |
| Age, y |  |  | |  |  |  |  |  |  |  |
|  | 65-69 | 66122 (29.96) | | 54.32 (53.73-54.91) | 70554 (32.03) | 54.59 (54.00-55.18) | 62300 (30.03) | 53.51 (52.86-54.15) | 66100 (29.19) | 62.59 (61.86-63.31) |
|  | 70-74 | 54909 (24.88) | | 59.00 (58.34-59.65) | 56740 (25.76) | 58.44 (57.77-59.11) | 57301 (27.62) | 60.25 (59.57-60.93) | 64705 (28.58) | 67.75 (66.98-68.51) |
|  | 75-79 | 43181 (19.57) | | 61.79 (61.05-62.53) | 41304 (18.75) | 61.72 (60.92-62.52) | 40475 (19.51) | 62.00 (61.09-62.90) | 46286 (20.44) | 71.10 (70.23-71.97) |
|  | ≥80 | 56476 (25.59) | | 63.68 (62.99-64.36) | 51699 (23.47) | 63.57 (62.84-64.30) | 47380 (22.84) | 63.96 (63.08-64.83) | 49331 (21.79) | 71.69 (70.75-72.62) |
| Sex |  |  | |  |  |  |  |  |  |  |
|  | male | 86702 (39.29) | | 58.84 (58.32-59.35) | 92133 (41.82) | 58.81 (58.29-59.33) | 91834 (44.27) | 59.34 (58.79-59.89) | 103302 (45.62) | 66.75 (66.15-67.33) |
|  | female | 133986 (60.71) | | 59.23 (58.80-59.65) | 128164 (58.18) | 58.76 (58.30-59.21) | 115622 (55.73) | 58.80 (58.27-59.32) | 123120 (54.38) | 68.16 (67.60-68.73) |
| Education |  |  | |  |  |  |  |  |  |  |
|  | ＜High School | 21945 (9.94) | | 54.35 (53.34-55.36) | 16824 (7.64) | 55.26 (54.14-56.38) | 12452 (6.00) | 55.08 (53.73-56.43) | 9978 (4.41) | 57.18 (55.46-58.89) |
|  | High School | 71601 (32.44) | | 58.33 (57.79-58.87) | 63116 (28.65) | 57.11 (56.51-57.70) | 52760 (25.43) | 56.14 (55.44-56.84) | 53428 (23.60) | 64.11 (63.35-64.88) |
|  | ＞High School | 127142 (57.61) | | 61.13 (60.70-61.57) | 140357 (63.71) | 60.65 (60.22-61.09) | 142244 (68.57) | 61.32 (60.85-61.78) | 163016 (72.00) | 70.95 (70.48-71.42) |
| Income level |  |  | |  |  |  |  |  |  |  |
|  | Less than $25,000 | 80652 (36.55) | | 55.02 (54.45-55.58) | 65376 (29.68) | 55.04 (54.41-55.67) | 53511 (25.79) | 54.49 (53.76-55.22) | 41531 (18.34) | 58.46 (57.50-59.42) |
|  | $25,000 to less than $50,000 | 73037 (33.10) | | 60.46 (59.90-61.02) | 70173 (31.85) | 58.81 (58.21-59.40) | 61780 (29.78) | 58.22 (57.52-58.93) | 70281 (31.04) | 66.27 (65.56-66.96) |
|  | $50,000 or more | 66999 (30.36) | | 62.44 (61.84-63.03) | 84748 (38.47) | 62.11 (61.55-62.67) | 92165 (44.43) | 63.04 (62.48-63.60) | 114610 (50.62) | 73.01 (72.45-73.57) |
| Race |  |  | |  |  |  |  |  |  |  |
|  | Non-Hispanic White | 193086 (87.49) | | 61.30 (60.98-61.62) | 192823 (87.53) | 60.52 (60.19-60.86) | 180515 (87.01) | 60.60 (60.22-60.97) | 197214 (87.10) | 69.82 (69.43-70.21) |
|  | Non-Hispanic Black | 11676 (5.29) | | 49.02 (47.62-50.41) | 11416 (5.18) | 50.10 (48.75-51.45) | 11228 (5.41) | 51.34 (49.87-52.80) | 12345 (5.45) | 59.30 (57.71-60.87) |
|  | Hispanic | 7262 (3.29) | | 49.19 (47.48-50.90) | 7602 (3.45) | 52.27 (50.58-53.95) | 6556 (3.16) | 53.62 (51.77-55.47) | 7357 (3.25) | 57.30 (55.16-59.41) |
|  | Other | 8664 (3.93) | | 55.67 (53.23-58.07) | 8456 (3.84) | 57.38 (54.70-60.03) | 9157 (4.41) | 57.28 (54.63-59.89) | 9506 (4.20) | 65.23 (62.65-67.71) |
