# Supplementary material for: Sociodemographic disparities in influenza vaccination among older adults in United States
Source: Front Public Health. 2025 Feb 7;13:1474677. doi: 10.3389/fpubh.2025.1474677 (PMC11843045; doi:10.3389/fpubh.2025.1474677)
Supplement: Supplementary file 2 [file Table_2.docx]

**Supplement table2 Age-standardized rates of** **influenza vaccination uptake varied in socioeconomic factors by 2011-2013,2014-2016,2017-2019 and 2020-2022.**

| Survey years | Races subgroups | Income level | | | | | |
| --- | --- | --- | --- | --- | --- | --- | --- |
|  |  |  | Less than $25,000 |  | $25,000 to less than $50,000 |  | $50,000 or more |
|  |  | Education level | Proportion (%) 95%CI |  | Proportion (%) 95%CI |  | Proportion (%) 95%CI |
| 2011-2013 |  |  |  |  |  |  |  |
|  | Non-Hispanic White |  |  |  |  |  |  |
|  |  | ＜High School | 57.15 (55.80-58.48) |  | 58.59 (56.35-60.80) |  | 59.37 (54.72-63.85) |
|  |  | High School | 57.96 (57.16-58.76) |  | 61.29 (60.41-62.17) |  | 60.19 (58.74-61.63) |
|  |  | ＞High School | 57.21 (56.29-58.13) |  | 63.27 (62.58-63.95) |  | 66.22 (65.59-66.84) |
|  | Non-Hispanic Black |  |  |  |  |  |  |
|  |  | ＜High School | 47.13 (44.10-50.18) |  | 48.21 (41.33-55.17) |  | 60.57 (49.12-70.97) |
|  |  | High School | 49.61 (46.87-52.37) |  | 47.34 (42.87-51.85) |  | 50.21 (40.65-59.75) |
|  |  | ＞High School | 46.63 (43.07-50.22) |  | 50.65 (45.90-55.39) |  | 53.87 (48.49-59.15) |
|  | Hispanic |  |  |  |  |  |  |
|  |  | ＜High School | 47.05 (44.23-49.89) |  | 57.49 (49.55-65.06) |  | 57.58 (41.33-72.35) |
|  |  | High School | 50.31 (46.37-54.25) |  | 55.05 (48.34-61.58) |  | 55.32 (45.66-64.61) |
|  |  | ＞High School | 46.13 (41.89-50.43) |  | 53.09 (47.57-58.54) |  | 51.39 (45.44-57.30) |
|  | Other |  |  |  |  |  |  |
|  |  | ＜High School | 52.04 (45.66-58.36) |  | 62.59 (54.57-69.97) |  | 68.64 (48.79-83.41) |
|  |  | High School | 47.98 (41.38-54.66) |  | 60.22 (53.76-66.34) |  | 61.60 (48.86-72.92) |
|  |  | ＞High School | 53.20 (47.25-59.06) |  | 58.29 (52.88-63.50) |  | 57.80 (52.32-63.09) |
| 2014-2016 |  |  |  |  |  |  |  |
|  | Non-Hispanic White |  |  |  |  |  |  |
|  |  | ＜High School | 55.54 (53.98-57.10) |  | 55.93 (53.30-58.52) |  | 54.24 (49.55-58.87) |
|  |  | High School | 56.66 (55.72-57.60) |  | 59.98 (59.04-60.91) |  | 59.58 (58.12-61.02) |
|  |  | ＞High School | 56.51 (55.46-57.57) |  | 61.34 (60.57-62.11) |  | 65.85 (65.24-66.47) |
|  | Non-Hispanic Black |  |  |  |  |  |  |
|  |  | ＜High School | 53.02 (49.88-56.13) |  | 54.84 (47.87-61.63) |  | 49.60 (33.51-65.76) |
|  |  | High School | 49.38 (46.43-52.33) |  | 48.32 (43.43-53.24) |  | 57.52 (49.70-64.99) |
|  |  | ＞High School | 44.51 (40.58-48.50) |  | 53.06 (49.60-56.49) |  | 53.41 (49.76-57.03) |
|  | Hispanic |  |  |  |  |  |  |
|  |  | ＜High School | 54.31 (51.41-57.17) |  | 45.63 (35.94-55.66) |  | 62.37 (47.34-75.34) |
|  |  | High School | 50.01 (45.83-54.18) |  | 51.20 (44.09-58.27) |  | 54.60 (45.30-63.59) |
|  |  | ＞High School | 49.20 (45.05-53.36) |  | 55.13 (49.80-60.35) |  | 55.07 (49.77-60.26) |
|  | Other |  |  |  |  |  |  |
|  |  | ＜High School | 60.63 (53.53-67.31) |  | 54.50 (43.39-65.18) |  | 62.68 (47.94-75.39) |
|  |  | High School | 57.05 (50.74-63.13) |  | 56.29 (47.98-64.26) |  | 64.20 (52.27-74.60) |
|  |  | ＞High School | 51.84 (46.03-57.60) |  | 56.58 (50.39-62.58) |  | 60.23 (54.86-65.37) |
| 2017-2019 |  |  |  |  |  |  |  |
|  | Non-Hispanic White |  |  |  |  |  |  |
|  |  | ＜High School | 54.15 (52.13-56.16) |  | 56.99 (53.89-60.03) |  | 54.31 (49.17-59.37) |
|  |  | High School | 54.94 (53.76-56.10) |  | 58.16 (56.99-59.23) |  | 59.62 (58.09-61.13) |
|  |  | ＞High School | 55.23 (54.05-56.42) |  | 60.75 (59.89-61.06) |  | 67.00 (66.36-67.63) |
|  | Non-Hispanic Black |  |  |  |  |  |  |
|  |  | ＜High School | 55.47 (51.94-58.95) |  | 54.51 (46.46-62.34) |  | 35.12 (23.69-48.56) |
|  |  | High School | 49.65 (46.61-52.69) |  | 50.98 (46.33-55.62) |  | 44.18 (34.63-54.18) |
|  |  | ＞High School | 51.98 (48.16-55.78) |  | 50.61 (46.82-54.38) |  | 56.06 (52.39-59.66) |
|  | Hispanic |  |  |  |  |  |  |
|  |  | ＜High School | 55.28 (51.86-58.64) |  | 57.98 (48.99-66.47) |  | 51.54 (37.87-64.98) |
|  |  | High School | 49.38 (44.40-54.37) |  | 50.04 (42.02-58.06) |  | 59.33 (51.37-66.83) |
|  |  | ＞High School | 53.39 (48.94-57.79) |  | 56.11 (50.06-61.99) |  | 59.42 (54.68-63.99) |
|  | Other |  |  |  |  |  |  |
|  |  | ＜High School | 50.40 (43.26-57.52) |  | 49.39 (40.93-57.88) |  | 38.58 (26.46-52.29) |
|  |  | High School | 55.29 (48.41-61.96) |  | 58.93 (48.19-68.88) |  | 64.67 (54.06-74.00) |
|  |  | ＞High School | 58.34 (52.70-63.76) |  | 52.89 (45.66-60.00) |  | 64.62 (60.05-68.95) |
| 2020-2022 |  |  |  |  |  |  |  |
|  | Non-Hispanic White |  |  |  |  |  |  |
|  |  | ＜High School | 57.89 (55.13-60.60) |  | 59.37 (56.04-62.62) |  | 61.34 (56.41-66.04) |
|  |  | High School | 60.44 (58.94-61.93) |  | 66.38 (65.26-67.48) |  | 68.96 (67.36-70.52) |
|  |  | ＞High School | 61.47 (59.97-62.94) |  | 69.76 (68.88-70.62) |  | 77.02 (76.45-77.58) |
|  | Non-Hispanic Black |  |  |  |  |  |  |
|  |  | ＜High School | 54.79 (49.61-59.87) |  | 58.91 (50.99-66.39) |  | 42.81 (29.82-56.86) |
|  |  | High School | 55.39 (51.34-59.36) |  | 62.28 (58.08-66.30) |  | 60.69 (53.87-67.13) |
|  |  | ＞High School | 56.99 (52.67-61.20) |  | 59.43 (56.06-62.71) |  | 64.84 (60.26-69.17) |
|  | Hispanic |  |  |  |  |  |  |
|  |  | ＜High School | 54.46 (50.17-58.70) |  | 54.36 (44.76-63.66) |  | 61.56 (44.20-76.40) |
|  |  | High School | 57.49 (52.89-61.96) |  | 61.83 (54.60-68.58) |  | 60.18 (48.27-71.00) |
|  |  | ＞High School | 52.04 (45.21-58.79) |  | 60.08 (54.05-65.83) |  | 69.08 (64.46-73.35) |
|  | Other |  |  |  |  |  |  |
|  |  | ＜High School | 49.66 (41.72-57.61) |  | 55.99 (45.03-66.14) |  | 55.86 (40.65-70.05) |
|  |  | High School | 63.62 (57.30-69.51) |  | 61.58 (53.75-68.86) |  | 62.81 (53.11-71.57) |
|  |  | ＞High School | 60.39 (54.23-66.24) |  | 68.47 (62.40-73.98) |  | 71.85 (67.35-75.94) |
