# Supplementary material for: Sociodemographic disparities in influenza vaccination among older adults in United States
Source: Front Public Health. 2025 Feb 7;13:1474677. doi: 10.3389/fpubh.2025.1474677 (PMC11843045; doi:10.3389/fpubh.2025.1474677)
Supplement: Supplementary file 3 [file Table_3.docx]

**Supplement table3 Statistical test parameters of joint associations of socioeconomic factors and races with influenza vaccination uptake**

|  |  | **Income level** | | | | | | | |
| --- | --- | --- | --- | --- | --- | --- | --- | --- | --- |
| **Races** | **Education** | Less than $25,000 | |  | $25,000 to less than $50,000 | |  | $50,000 or more |  |
|  |  | Std. error | t value |  | Std. error | t value |  | Std. error | t value |
| Non-Hispanic White |  |  |  |  |  |  |  |  |  |
|  | ＜High School | 0.047 | 3.33 |  | 0.058 | 4.76 |  | 0.078 | 3.36 |
|  | High School | 0.046 | 4.82 |  | 0.055 | 9.47 |  | 0.060 | 10.37 |
|  | ＞High School | 0.047 | 5.09 |  | 0.060 | 12.38 |  | 0.079 | 20.10 |
| Non-Hispanic Black |  |  |  |  |  |  |  |  |  |
|  | ＜High School | refer. |  |  | 0.102 | 0.63 |  | 0.130 | -1.56 |
|  | High School | 0.046 | -1.33 |  | 0.062 | 0.88 |  | 0.103 | 0.69 |
|  | ＞High School | 0.050 | -1.51 |  | 0.057 | 1.60 |  | 0.067 | 5.09 |
| Hispanic |  |  |  |  |  |  |  |  |  |
|  | ＜High School | 0.053 | 1.24 |  | 0.117 | 1.38 |  | 0.292 | 2.47 |
|  | High School | 0.060 | 0.21 |  | 0.096 | 1.90 |  | 0.153 | 1.90 |
|  | ＞High School | 0.058 | -1.00 |  | 0.088 | 2.70 |  | 0.088 | 5.39 |
| Other |  |  |  |  |  |  |  |  |  |
|  | ＜High School | 0.092 | 0.55 |  | 0.169 | 0.75 |  | 0.438 | 0.75 |
|  | High School | 0.100 | 2.75 |  | 0.147 | 2.71 |  | 0.260 | 2.73 |
|  | ＞High School | 0.089 | 2.59 |  | 0.113 | 4.85 |  | 0.113 | 9.03 |
